# Supplementary material for: Content-rich biological network constructed by mining PubMed abstracts
Source: BMC Bioinformatics. 2004 Oct 8;5:147. doi: 10.1186/1471-2105-5-147 (PMC528731; doi:10.1186/1471-2105-5-147)
Supplement: Additional File 2 — The original results of the above study (non-essential files are deleted to keep the file size under the limit set by BMC bioinformatics). [file 1471-2105-5-147-S2.bz2 › chilibotAdditionalFile2/dip30/424ID11470772E10354/html/CCL1_CCR8.html]

 


 **CCL1** and **CCR8** 
  
Found 39 abstracts in PubMed, retrieved 30.  
 

 What does Google say? 
 PDF only 
| .edu only 

---

**Interactive relationship** (e.g. stimulation, inhibition, etc)

**Neutral relationship**- Further analysis demonstrated that  **CCL1**  activates the MAPK pathway in  **CCR8**  transfected CHO cells.  Ref: 12645948 Eur J Immunol, 2003
- ... interactions of CCR4 with MDC CCL22 or TARC CCL17,  **CCR8**  with I 309  [ **CCL1** ]   **CCL1** , and CRTH2 with prostaglandin D 2 play a critical role in the allergen induced recruitment of Th2 cells in the target tissues of allergic inflammation.  Ref: 12009564 Mol Immunol, 2002
- These results provide strong evidence that, at physiologically relevant concentrations, I 309  [ **CCL1** ]  is the only known human ligand for  **CCR8** .  Ref: 10540332 Eur J Immunol, 1999
- Th2 cells preferentially express  **CCR8**  and migrate selectively to its ligand, CC chemokine ligand CCL 1  [ **CCL1** ] .  Ref: 12707363 J Immunol, 2003
- **CCR8**  is the sole receptor for the human CC chemokine I 309  [ **CCL1** ] , as well as for viral monocyte inflammatory protein I vMIP I, a human chemokine homologue induced in human cells by the Kaposi sarcoma related human herpesvirus 8.  Ref: 11133740 Blood, 2001
- The role of CCRS was supported by the observation that vMIP I, another  **CCR8**  ligand identified from the genome of a T cell transforming herpes virus, shared  **CCL1**  anti apoptotic activity.  Ref: 12645948 Eur J Immunol, 2003
- An examination of the I 309  [ **CCL1** ]  structure will aid in an understanding of the specificity of this protein with its receptor,  **CCR8** .  Ref: 10821677 Biochemistry, 2000
- **CCR8**  is detected at later time points than CCR4 and exclusively on differentiated Th2 cells as revealed by analysis of mRNA expression and responsiveness to  **CCL1** .  Ref: 11981813 Eur J Immunol, 2002
- The CC chemokine receptor CCR 8  [ **CCR8** ]  belongs to the seven transmembrane spanning receptor family and functionally responds to the eukaryotic CC chemokines I 309  [ **CCL1** ] , thymus and activation regulated chemokine, macrophage inflammatory protein 1 beta MIP 1b and to the products viral MIP I and viral MIP II of the Kaposi associated herpesvirus HHV 8.  Ref: 10611408 Forum Genova < MedlineTA>Forum Genova , 2002
- In vitro polarized human Th2 cells preferentially express the chemokine receptors CCR3, CCR4, and  **CCR8**  and migrate to their ligands eotaxin, monocyte derived chemokine MDC, thymus and activation regulated chemokine TARC, and I 309  [ **CCL1** ] .  Ref: 11390417 J Clin Invest, 2001
- Three human chemokines I 309  [ **CCL1** ] , thymus and activation regulated chemokine TARC and macrophage inflammatory protein 1beta MIP 1beta have been reported to be ligands for CC chemokine receptor 8  [ **CCR8** ]   **CCR8** .  Ref: 10540332 Eur J Immunol, 1999
- Treg cells specifically express the chemokine receptors CCR4 and  **CCR8**  and represent a major subset of circulating CD4 T cells responding to the chemokines macrophage derived chemokine MDC CCL22, thymus and activation regulated chemokine TARC CCL17, I 309  [ **CCL1** ]   **CCL1** , and to the virokine vMIP I ligands of CCR4 and  **CCR8** .  Ref: 11560999 J Exp Med, 2001
- T regulatory cells, which inhibit dendritic cell function and are probably involved in the termination of ACD, are sensitive to MCP 1, MIPs and TARC, but express high levels of  **CCR8**  and are more specifically attracted by I 309  [ **CCL1** ] .  Ref: 11876523 Arch Dermatol Res, 2002
- vMIP I and I 309  [ **CCL1** ]  appear to act as  **CCR8**  agonists binding to and inducing cytosolic Ca 2 elevation through the receptor.  Ref: 10419462 J Biol Chem, 1999
- The chemokine receptor  **CCR8**  mediates human endothelial cell chemotaxis induced by I 309  [ **CCL1** ]  and Kaposi sarcoma herpesvirus encoded vMIP I and by lipoprotein a stimulated endothelial cell conditioned medium.  Ref: 11133740 Blood, 2001
- Increased responsiveness of murine eosinophils to MIP 1beta CCL4 and TCA 3  [ **CCL1** ]   **CCL1**  is mediated by their specific receptors, CCR5 and  **CCR8** .  Ref: 12050188 J Leukoc Biol, 2002

**Non-interactive relationship** (e.g. studied together, co-existance, homology, etc.)

- **CCR8** , the  **CCL1**  receptor, was identified on endothelial cells, and  **CCL1**  was found to be a chemoattractant for these cells.  Ref: 12489497 Isr Med Assoc J, 2002
- TCR triggering of Th1 and Th2 cells leads to production of MDC and I 309  [ **CCL1** ]  CCR4 and  **CCR8**  ligands, respectively.  Ref: 10760790 Eur J Immunol, 2000
- To determine whether human immunodeficiency virus type 1 HIV 1 coreceptors besides CXCR4 and CCR5 are involved in HIV 1 infection of the thymus, we focused on  **CCR8** , a receptor for the chemokine I 309  [ **CCL1** ] , because of its high expression in the thymus.  Ref: 10888633 J Virol, 2000
- The aim of our study was to characterize the role of the CC chemokine receptor 8  [ **CCR8** ]   **CCR8** , the only described receptor for  **CCL1** , in the rescue of murine thymic lymphoma cells and murine thymocytes from dexamethasone dex induced apoptosis.  Ref: 12525579 J Leukoc Biol, 2003
- The most active chemokines for resting Th IL 10, in terms of calcium mobilization and in vitro migration, were in order of potency CCL2 monocyte chemoattractant protein 1, CCR2 ligand, CCL4 macrophage inflammatory protein 1beta, CCR5 ligand, CCL3 macrophage inflammatory protein 1alpha, CCR1 5 ligand, CCL17 thymus and activation regulated chemokine, CCR4 ligand,  **CCL1**  I 309  [ **CCL1** ] ,  **CCR8**  ligand, CXCL12 stromal derived factor 1, CXCR4, and CCL11 eotaxin, CCR3 ligand.  Ref: 11145678 J Immunol, 2001
- Th2 lymphocytes, which are critical for allergy, employ the CC chemokine receptors CCR4 and  **CCR8**  with the ligands thymus and activation regulated chemokine TARC, macrophage derived chemokine MDC and I 309  [ **CCL1** ] , respectively.  Ref: 11340325 Int Arch Allergy Immunol, 2001
- I 309  [ **CCL1** ]  and  **CCR8**  mRNAs were not detected in unaffected skin and were up regulated at the skin site of nickel allergic reaction, with an earlier expression kinetics compared with IL 10 and IL 4.  Ref: 11145678 J Immunol, 2001
- Inhibition of I 309  [ **CCL1** ]  receptor  **CCR8**  signaling by pertussis toxin increased the apoptosis rate of ATL cell cultures in the presence and absence of external apoptotic stimuli.  Ref: 11493464 Blood, 2001
- Suppression of I 309  [ **CCL1** ]  secretion results in a decreased chemotactic effect on L1.2 cells transfected with human  **CCR8** , indicating that IL 12 and IFN alpha may inhibit the recruitment of  **CCR8**  expressing cells such as Th2 cells.  Ref: 10760790 Eur J Immunol, 2000
- Such pattern of  **CCL1**  responsiveness suggests that the  **CCR8**   **CCL1**  axis may regulate trafficking of cutaneous Treg and memory T cells into the skin.  Ref: 12442333 Eur J Immunol, 2002
- We studied the role of the  **CCR8**  ligand,  **CCL1** , in the specific recruitment of Th2 cells and eosinophils to the lung in a murine model of allergic airway disease.  Ref: 12707363 J Immunol, 2003
- **CCR8**  I 309  [ **CCL1** ]  driven recruitment of both resting and activated Th IL 10 cells may be critically involved in the regulation of Th1 mediated skin allergic disorders.  Ref: 11145678 J Immunol, 2001
- Although it has been hypothesized that the rescuing effect of  **CCL1**  from apoptosis could be  **CCR8**  mediated, here, we formally demonstrate the role of such receptor as its selective antagonist encoded by the MC148 gene of molluscum contagiosum virus MC148 vMCC I inhibits v MIP 1 and  **CCL1**  induced rescue activity.  Ref: 12525579 J Leukoc Biol, 2003
- The  **CCR8**  ligand, TCA3  [ **CCL1** ] , is shown to be produced predominantly by resident microglia of the CNS in response to TNF.  Ref: 12471141 J Immunol, 2002
- Polyclonal and monoclonal anti I 309  [ **CCL1** ]  antibodies as well as an antibody against a portion of the extracellular domain of  **CCR8** , the I 309  [ **CCL1** ]  receptor, inhibited the increase in monocyte chemotactic activity induced by apo a.  Ref: 10942748 Circulation, 2000
- Using antibodies specific for CCR5 and  **CCR8** , the chemotactic response to MIP 1beta and TCA 3  [ **CCL1** ] , respectively, was reduced significantly.  Ref: 12050188 J Leukoc Biol, 2002
- Because of its pharmacological selectivity, the MC148 protein could be a useful tool in the delineation of the role played by  **CCR8**  and its endogenous ligand, I 309  [ **CCL1** ] .  Ref: 10620615 J Exp Med, 2000
- Here, we show that human peripheral blood CD4 and CD8 cutaneous CLA, but not intestinal memory integrin beta 7 nor IL 4 producing T cells, represent major subpopulations of circulating T cells that specifically migrate in response to the chemokine I 309  [ **CCL1** ]   **CCL1**  by virtue of  **CCR8**  expression.  Ref: 12442333 Eur J Immunol, 2002
- We show here that the  **CCR8**  restricted agonist Kaposi sarcoma associated herpesvirus encoded chemokine viral macrophage inflammatory protein 1 vMIP 1 rescues thymic lymphoma cells from dex induced apoptosis, similar to  **CCL1** ,.  Ref: 12525579 J Leukoc Biol, 2003
- Additionally, increased expression of the  **CCR8**  ligand, TCA 3  [ **CCL1** ] , has been detected in affected lungs in a mouse model of asthma.  Ref: 12496446 J Immunol, 2003
- Using  **CCR8**  mice, evidence is provided that TCA3  [ **CCL1** ]   **CCR8**  interactions contribute to rapid onset CNS inflammation.  Ref: 12471141 J Immunol, 2002
- Immunohistochemical studies identified  **CCR8**  and I 309  [ **CCL1** ]  on the endothelium of human atherosclerotic plaques and in endothelial derived spindle cells of Kaposi sarcoma.  Ref: 11133740 Blood, 2001
- In calcium mobilization assays, MC148 had no effect on its own on any of the chemokine receptors, but in a dose dependent manner blocked the stimulatory effect of the endogenous I 309  [ **CCL1** ]  chemokine on  **CCR8**  without affecting chemokine induced signaling of any other receptor.  Ref: 10620615 J Exp Med, 2000
